# Observation of risk factors for shoulder subluxation after stroke using ultrasonography to measure thickness of the supraspinatus muscle: a cross-sectional study

**DOI:** 10.3389/fneur.2025.1532004

**Published:** 2025-03-19

**Authors:** Yu Qu, Xiue Shi, Yongyong Wang, Tiecheng Ji, Lei Chen, Suli Yu, Ming Huo

**Affiliations:** ^1^Qilu Hospital of Shandong University, Jinan, Shandong, China; ^2^School of Rehabilitation Science and Engineering, University of Health and Rehabilitation Sciences, Qingdao, Shandong, China; ^3^Shaanxi Provincial Rehabilitation Hospital, Xi'an, Shaanxi, China; ^4^Jilin Province Power Hospital, Changchun, Jilin, China; ^5^Qingdao Hospital, University of Health and Rehabilitation Sciences (Qingdao Municipal Hospital), Qingdao, Shandong, China

**Keywords:** shoulder subluxation, risk factors, stroke, ultrasonography, supraspinatus thickness

## Abstract

**Background:**

The prevention of shoulder subluxation, which is mainly caused by stroke, remains a challenge in rehabilitation treatment. While shoulder subluxation is a common problem after stroke, adequate objective predictors is lacking.

**Aim:**

This study aimed to determine the acromion-greater tuberosity (A-GT) distance using ultrasound imaging in stroke patients, investigate the risk factors for shoulder subluxation after stroke, analyze the etiology of shoulder subluxation, and effectively prevent its occurrence.

**Design:**

Cross-sectional study.

**Setting:**

Inpatient rehabilitation unit.

**Population:**

One hundred twenty-eight patients in our hospital between 2023 and 2024 with a confirmed diagnosis of stroke (age 59.1 ± 10.5 years; range 22–82 years; 82 males, 46 females; 100 cases of cerebral infarction and 28 of cerebral hemorrhage; 79 cases of left stroke and 49 of right; 82 patients in stage I, 19 in stage II; 11 in stage III, 9 in stage IV, and 7 in stage V).

**Methods:**

Ultrasonographic variables (A-GT distance and supraspinatus thickness on the lesion side) were collected. The paired *t*-test was adopted to compare the A-GT distance and supraspinatus thickness between the paralyzed and non-paralyzed sides. Data (A-GT distance, supraspinatus thickness on the lesion side) were analyzed using one-way analysis of variance (ANOVA) and multiple comparison tests. Spearman’s correlation and multivariate linear regression were used to analyze the associations between the A-GT distance and specific clinical characteristics.

**Results:**

The A-GT distance was significantly increased in the paralyzed sides compared with the non-paralyzed sides (*p* < 0.01; paired *t*-test). The supraspinatus thickness was significantly reduced in the paralyzed compared sides with the non-paralyzed sides (*p* < 0.01; paired *t*-test). Significant differences were observed in A-GT distance between the sex, stroke type, and Brunnstrom stage groups (*p* < 0.01). Supraspinatus thickness on the lesion side showed significant differences between the sex, type of stroke, and lesion side groups (*p* < 0.01). A correlation between A-GT distance and supraspinatus thickness was also found (*r* = −0.474, *p* < 0.01). Based on the multivariate regression analysis, the independent risk factors for shoulder subluxation after stroke included type of stroke, supraspinatus thickness on the lesioned side, and Brunnstrom stage.

**Conclusion:**

Acromio-greater tuberosity distance and reduced supraspinatus thickness on ultrasound, low Brunnstrom stage and history of cerebral hemorrhage were all found to be significant risk factors for shoulder subluxation after stroke.

**Clinical rehabilitation impact:**

We investigated the risk factors for shoulder subluxation after stroke, which may help physicians understand its mechanism and prevent its occurrence.

## Introduction

Stroke is a leading cause of death and disability worldwide. Approximately 15 million people suffer a stroke annually, having a devastating impact on society and the healthcare system (1). Shoulder subluxation is a general term for glenohumeral subluxation on the lesioned side after a stroke (2). Shoulder subluxation is observed in 17–64% of patients with hemiplegia after a stroke, particularly within the initial 3 weeks following the onset of hemiplegia (3). This causes poor functional recovery, prolonged hospitalization, and reduced quality of life in patients with stroke (4, 5).

The shoulder is the largest and most complex joint in the human body. Shoulder subluxation is usually considered a partial or incomplete dislocation of the humeral head from the glenoid fossa, or a translation between the humeral head and glenoid fossa (6, 7). Size mismatch between the humeral head, which is quite large, and the glenoid, which is small, render proprioception especially critical in the shoulder, sacrificing stability for increased ROM (8). The head of the humerus tends to dislocate anteriorly and inferiorly due to weakness of the rotator cuff muscles or laxity of the glenohumeral ligaments (9, 10).

Previous studies have investigated the potential factors associated with shoulder subluxation in patients with stroke. Fifty percent of subjects with hemorrhagic stroke are prone to subluxation, whereas only one-third of patients with ischemic stroke develop malalignment (11). The pathogenesis of shoulder subluxation is mostly unknown and is considered multifactorial. Proprioceptive deficits, hemorrhagic stroke, lower motor recovery, flaccidity, mishandling, poor positioning, and influence of gravitational pull are important risk factors for subluxation (9, 12). Given the sizable difference between the humeral head and the glenoid, shoulder mobility is limited to three directions: flexion and extension, adduction and abduction, and internal and external rotation. However, it loses stability (2). The bony structures and ligaments provide static stability, whereas the rotator cuff muscle groups and shoulder girdle muscles provide dynamic stability (13). However, the flaccid paralysis period after stroke and the paralysis of the supraspinatus and deltoid muscles cannot fix the humeral head in the superficial glenoid, causing an increase in acromion-greater tuberosity (A-GT) distance (12). Spasticity of the internal rotation muscles (latissimus dorsi, pectoralis major, and subscapularis) results in the head of the humerus tilting backward, causing posterior shoulder subluxation (14). Nevertheless, the association between shoulder subluxation and many of these factors remains controversial.

Previous studies have indicated that palpation methods for assessing shoulder subluxation are less sensitive in detecting early signs of Glenohumeral subluxation (GHS) and mild subluxation linked to severe paralysis (15). While roentgenographic evaluation is objective, reliable, and effective (16, 17), it is seldom used in clinical practice due to concerns about cost, time, and radiation exposure. Recently, ultrasound imaging has emerged as a useful tool for evaluating shoulder subluxation. By measuring the AGT distance, clinicians can determine the presence of subluxation in hemiplegic patients (18). Ultrasound measurement of the AGT distance demonstrates adequate intra- and inter-rater reliability and has proven to be highly reliable among both experienced and novice evaluators (19, 20).

This cross-sectional study aimed to determine the risk factors for subsequent shoulder subluxation during stroke rehabilitation. Age, sex, lesioned side, type of stroke, thickness of the supraspinatus on the lesioned side, and Brunnstrom recovery stage (BRS) were considered.

## Materials and methods

### Participants and setting

A total of 128 patients with hemiplegia who were rehabilitated at the Jilin City Puren Hospital of Traditional Chinese Medicine between 2023 and 2024 were enrolled in this cross-sectional study. All the participants provided informed consent to participate in this study. The inclusion criteria were ischemic and hemorrhagic stroke; patients of all ages were included regardless of sex, lesioned side and phase of recovery. The exclusion criteria were traumatic shoulder subluxation or shoulder surgery before stroke onset, hemiparesis due to head injury, severe cognitive impairment, and an unstable medical condition.

### Physical examination

Variables included demographic, clinical, and ultrasonographic characteristics. Age, sex, the lesioned side, stroke type, supraspinatus thickness on the lesioned side, and Brunnstrom motor recovery stage (B-stage) of the arms and hands were recorded. B-stage is a valid and reliable method for assessing motor functions consisting of six stages: (I) flaccid paralysis; (II) involuntary movement and spasticity in a synergy pattern; (III) increased spasticity and voluntary control in a synergy pattern; (IV) decreased spasticity and voluntary movement without a synergy pattern; (V) low spasticity and more complex movements; and (VI) no spasticity and normal movements (21, 22).

### Sonographic examination

Ultrasonographic evaluations were performed using a 7.5 MHz linear array probe (Sonosite180Plus, United States). Ultrasound imaging has long been recognized as a non-invasive method for quantifying muscle shape and contraction, and is widely used as a research and clinical tool throughout the rehabilitation process. The patients were held in a sitting position (with their shoulder in a neutral position, elbow flexed at 90°, and forearm in pronation), whereas the A-GT distance and supraspinatus thickness were measured (23). The ultrasound probe was placed vertically at the midpoint of the scapula and moved parallel to the thickest part of the supraspinatus muscle, and in longitudinal imaging, the vertical distance between the upper and lower edges of the muscle at the scapular notch was measured. The distance between the acromion and the upper part of the greater tuberosity of the humerus was measured using ultrasonography (20). All the measurements were performed by the same physician.

### Statistical analysis

Statistical analysis was performed using the SPSS version 26.0 software. Data was expressed in mean ± standard deviation (SD). The paired t-test was adopted to compare the A-GT distance and supraspinatus thickness between the paralyzed and non-paralyzed sides. One-way analysis of variance (ANOVA) and multiple comparisons (Bonferroni test) were used to test for statistically significant differences in the A-GT distance and supraspinatus thickness on the lesioned side with different factors. Spearman correlation coefficients were used to analyze the relationships between such factors and the A-GT distance measurements. Statistical significance was set at *p* < 0.05. The following method was used: “Linear multiple regression: Fixed model, R2 deviation from zero.” The A-GT distance was used as the dependent variable.

## Results

Patient demographic, clinical, and ultrasonographic characteristics are summarized in Table 1. A total of 128 patients (82 males and 46 females) were included in this study. Among these, 79 had left hemiplegia, and 49 had right hemiplegia. Although 100 patients experienced cerebral infarctions, 28 had cerebral hemorrhages. Stage 1 was present in 82 (64.1%) patients, stage 2 in 19 (14.8%), stage 3 in 11 (8.6%), stage 4 in 9 (7.0%), and Stage 5 in 7 (5.5%).

**Table 1:** Patient demographic, clinical, and ultrasonographic measurements.

		*N* (%)/Mean ± SD
	Age (years)	59.1 ± 10.5
	Supraspinatus thickness (cm)	1.82 ± 0.37
	A-GT distance (cm)	1.70 ± 0.46
Sex	Male	82 (64.1)
	Female	46 (35.9)
Lesioned side	Left	79 (61.7)
	Right	49 (38.3)
Stroke Type	Cerebral infarction	100 (78.1)
	Cerebral hemorrhage	28 (21.9)
Brunnstrom stage	Stage 1	82 (64.1)
	Stage 2	19 (14.8)
	Stage 3	11 (8.6)
	Stage 4	9 (7.0)
	Stage 5	7 (5.5)
Number of months since the stroke (months)		1.43 ± 1.44

SD, standard deviation; A-GT: acromion-greater tuberosity distance.

**Figure 1:**
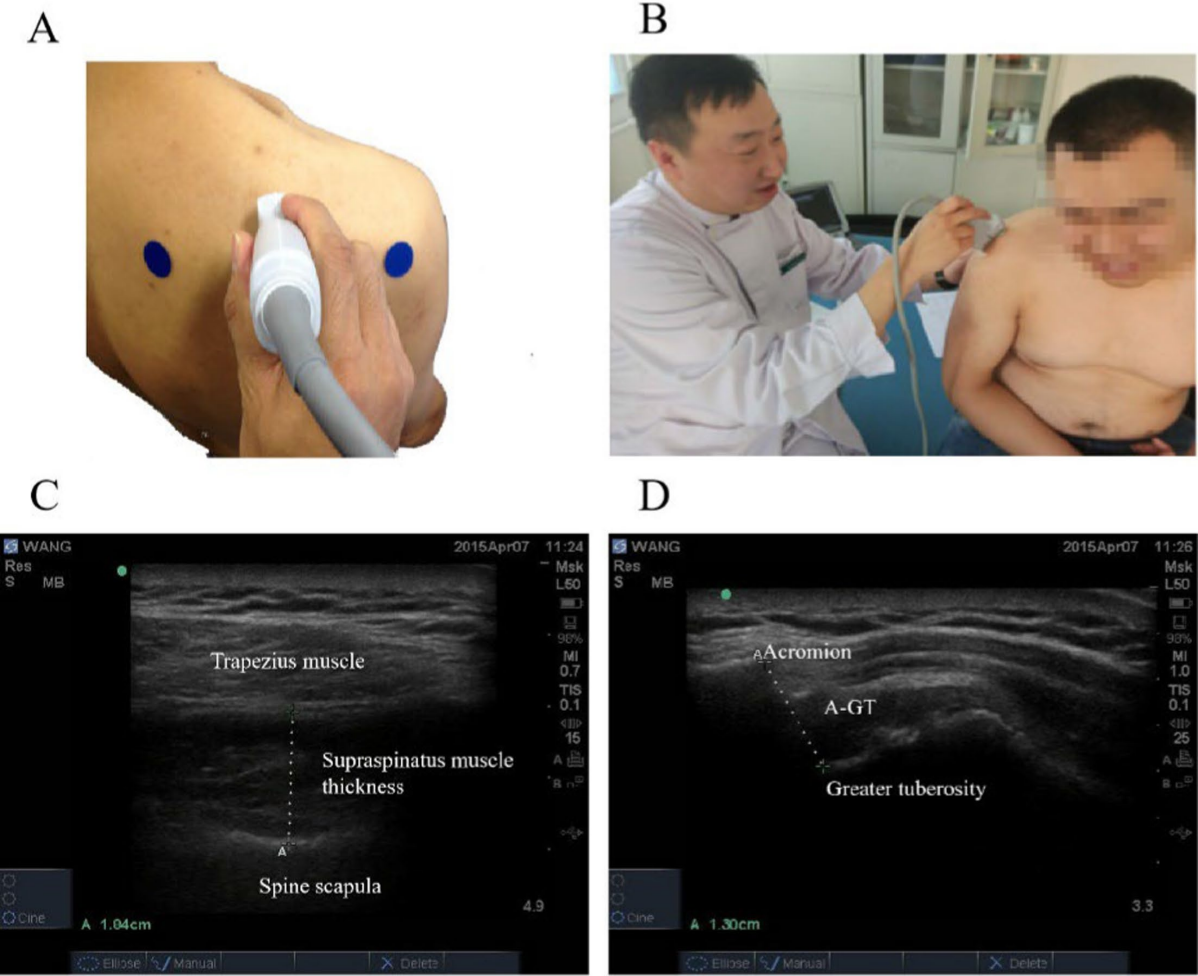
The supraspinatus muscle thickness, AGT were measured by ultrasound imaging. **(A)** The ultrasound probe was placed vertically at the midpoint of the scapula and moved parallel to the thickest part of the supraspinatus muscle. **(B)** The ultrasound probe was placed vertically between the acromion and the upper part of the greater tuberosity of the humerus. **(C)** Supraspinatus muscle thickness. **(D)** Acromion-greater tuberosity distance.

The supraspinatus muscle thickness, AGT were measured by ultrasound imaging in Figure 1. The ultrasound probe was placed vertically at the midpoint of the scapula and moved parallel to the thickest part of the supraspinatus muscle to measure the thickness of supraspinatus muscle; The ultrasound probe was placed vertically to measure the distance between the acromion and the upper part of the greater tuberosity of the humerus.

Table 2 demonstrate a comparison of the A-GT distance and supraspinatus thickness between the paralyzed and non-paralyzed sides. The A-GT distance was significantly increased in the paralyzed sides compared with the non-paralyzed sides (*p* < 0.01; paired *t*-test). The supraspinatus thickness was significantly reduced in the paralyzed compared sides with the non-paralyzed sides (*p* < 0.01; paired *t*-test).

**Table 2:** Comparison of the A-GT distance and supraspinatus thickness between the paralyzed and non-paralyzed sides.

	Paralyzed sides	Non-paralyzed sides	*p*-value
A-GT distance (SD)	1.71 (0.47)	1.28 (0.24)	0.00
Supraspinatus thickness (SD)	1.83 (0.39)	1.97 (0.36)	0.00

All tests of significance were performed using paired *t*-test. All table values are displayed as mean (standard deviation). SD, standard deviation.

The correlations between sonographic examinations and clinical findings are summarized in Table 3. The supraspinatus thickness on the lesioned side and the A-GT distance were negatively correlated (Spearman rho = −0.474, *p* < 0.01). In contrast, sex and A-GT distance were positively correlated (Spearman’s rho = 0.372, *p* < 0.01). Similarly, stroke type and A-GT distance were positively correlated (Spearman’s rho = 0.312, *p* < 0.01). Finally, BRS and A-GT distance were negatively correlated (Spearman’s rho = −0.382, *p* < 0.01). The A-GT distance did not correlate with age and lesioned side (*p* > 0.05).

**Table 3:** Factors associated with hemiplegic shoulder subluxation (*n* = 128).

	A-GT distance	Age	Supraspinatus thickness	Sex	Stroke Type	Lesioned side	Brunnstrom stage
A-GT distance	1.000						
Age	−0.039	1.000					
Supraspinatus thickness	−0.474**	−0.130	1.000				
Sex	0.372**	0.196*	−0.420**	1.000			
Stroke Type	0.312**	−0.103	−0.262**	0.155	1.000		
Lesioned side	−0.105	−0.075	0.333**	−0.087	−0.028	1.000	
Brunnstrom stage	−0.382**	0.088	0.184*	−0.119	−0.044	−0.042	1.000

**p < 0.01, *p < 0.05.

Sex: male/female; lesioned side: left/right; stroke type: cerebral infarction/cerebral hemorrhage.

Brunnstrom stage: stage 1/stage 2/stage 3/stage 4/stage 5.

The results of the one-way ANOVA for the A-GT distance showed that A-GT distance was greater in females than in males with hemiplegia, greater in patients with cerebral hemorrhage than in patients with cerebral infarction, and greater in BRS 1 than in BRS 3, and BRS 1 than in BRS 4 (*p* < 0.01). The difference in A-GT distance observed between patients with left and right hemiplegia was not statistically significant (Tables 4–6).

**Table 4:** One-way ANOVA for A-GT distance by factor (*n* = 128).

Variables			*p-*value
Sex	Male	1.60 ± 0.44	**Female > Male
	Female	1.89 ± 0.46	
Lesioned side	Left	1.76 ± 0.51	
	Right	1.62 ± 0.38	
Stroke Type	Cerebral infarction	1.60 ± 0.34	**Cerebral hemorrhage > Cerebral infarction
	Cerebral hemorrhage	2.08 ± 0.65	
Brunnstrom stage	Stage 1	1.83 ± 0.51	**Stage 1 > stage 3, **stage 1 > stage4
	Stage 2	1.61 ± 0.31	
	Stage 3	1.37 ± 0.26	
	Stage 4	1.37 ± 0.19	
	Stage 5	1.49 ± 0.26	

Values are means ± standard deviation. **p < 0.01.

Sex: male/female; lesioned side: left/right; stroke type: cerebral infarction/cerebral hemorrhage.

Brunnstrom stage: stage 1/stage 2/stage 3/stage 4/stage 5.

**Table 5:** One-way ANOVA for supraspinatus thickness on the lesioned side by factor (*n* = 128).

Variables			*p-*value
Sex	Male	1.94 ± 0.38	**Female < Male
	Female	1.62 ± 0.28	
Lesioned side	Left	1.75 ± 0.36	**Left < Right
	Right	1.96 ± 0.38	
Stroke type	Cerebral infarction	1.88 ± 0.38	**Cerebral hemorrhage < Cerebral infarction
	Cerebral hemorrhage	1.64 ± 0.33	
Brunnstrom stage	Stage 1	1.78 ± 0.36	
	Stage 2	1.80 ± 0.33	
	Stage 3	1.89 ± 0.54	
	Stage 4	2.09 ± 0.38	
	Stage 5	2.03 ± 0.17	

Values are means ± standard deviation. **p < 0.01.

Sex: male/female; lesioned side: left/right; stroke type: cerebral infarction/cerebral hemorrhage.

Brunnstrom stage: stage 1/stage 2/stage 3/stage 4/stage 5.

**Table 6:** Multivariate linear regression model of hemiplegic shoulder subluxation after a stroke (*n* = 128).

Variables	Beta	*t*	*p*-value	95% CI^a^	
Constant		13.863	<0.01**	2.109	2.812
Supraspinatus thickness (cm)	−0.360	−3.802	<0.01**	−0.548	−0.173
Stroke type	0.380	4.528	<0.01**	0.214	0.546
Brunnstrom stage	−0.102	−3.559	<0.01**	−0.159	−0.045

**p < 0.01.

Stroke type: cerebral infarction/cerebral hemorrhage; Brunnstrom stage: stage 1/stage 2/stage 3/stage 4/stage 5.

The results of the one-way ANOVA for the A-GT distance showed that the supraspinatus thickness on the lesioned side was smaller in females than in males with hemiplegia, lower in left compared to right hemiplegic patients, and thinner in patients with cerebral hemorrhage than in those with cerebral infarction.

The results of the multiple linear regression showed that the factors influencing the A-GT distance included the supraspinatus thickness of the lesioned side, stroke type, and BRS (*R*^2^ = 0.360, *p* < 0.01). No significant results were instead reported for age, sex, and lesioned side.

## Discussion

Shoulder stability includes static and dynamic components. Static structures include skeletal structures, intra-articular relative pressures, capsular ligamentous structures, and the glenohumeral labrum. Dynamic aspects include coordinated muscle contraction around the joint and regulation by the neuromuscular system (24). Proprioception relies on mechanoreceptors in the capsuloligamentous structures of the shoulder joint, including the Ruffini and Pacinian-like corpuscles, free nerve endings, and the Golgi apparatus. Proprioceptive dysfunction is correlated with higher rates of musculoskeletal injury, recurrence, and persistence of disabilities such as shoulder subluxation.

Patients with stroke and severe paralysis reported shoulder subluxation more frequently during rehabilitation. The etiology of glenohumeral subluxation is complex and related to denervation of the supraspinatus and deltoid muscles as a result of brain injury (25); detachment of the humeral head from the glenoid under the influence of gravity, poor trunk control on the lesioned side affecting the direction of scapular movement, tension contracture of the pectoralis major and latissimus dorsi muscles (shoulder adductors), which changes the direction of the glenoid, synergistic effect of excessive adduction and internal rotation that aggravates the subluxation of the shoulder joint, violent pulling on the arm of the affected side, and lack of good limb positioning (26). Decreased rotator cuff muscle strength and shoulder instability can lead to diminished proprioception of the shoulder joint, further contributing to shoulder subluxation (27).

Additionally, compared to patients with shoulder pain but without shoulder subluxation, those with shoulder subluxation may be more susceptible to a number of injuries, such as long head tendon-glenoid labral injuries, glenoid labral injuries, and bone marrow edema (28). Furthermore, hemiplegic shoulder pain (HSP) can induce various complications such as peripheral soft tissue involvement, bicipital tendonitis, subacromial impingement, sensory loss, and shoulder-hand syndrome (29–34). These symptoms result in shoulder pain and do not allow any type of voluntary movement of the affected shoulder. Thus, they are associated with a decline in the patient’s quality of life and a complication for the motor function recovery (35).

At present, the rehabilitation guidelines for the subluxation therapy include normal limb positioning by counteracting the gravitational pull, shoulder support (to realign the humeral head in the glenoid), although it has inadequate evidence for its effectiveness (36, 37), neuromuscular electrical stimulation (NMES), and functional electrical stimulation (FES) by activating the paretic rotator cuff muscle groups to prevent shoulder subluxation only during the acute stage of stroke (38–40). Sling training of the rotator cuff muscle group to strengthen the muscles can reduce subluxation. Currently, methods to improve shoulder subluxation are not clinically available, and long-term positive outcomes are not possible (41–44). Once shoulder subluxation occurs, the biomechanical-level pathophysiology does not provide a structural basis for neuro-motor recovery (45–47). Therefore, it is necessary to identify the risk factors for shoulder subluxation for early prevention after stroke.

There are several methods for measuring the A-GT distance in clinical practice. The finger width method grades subluxation but lacks the sensitivity to detect early signs of glenohumeral or minor subluxations (distances <0.5 cm). X-rays show the severity of subluxation in millimeters; however, problems related to the cost, time involved, and patient exposure to radiation have been identified (48). In this study, ultrasonography was used as an accurate and sensitive method for assessing subluxation and supraspinatus thickness.

This study aimed to identify the risk factors associated with shoulder subluxation in patients with stroke. To the best of our knowledge, few studies on shoulder subluxation in patients with stroke that consider clinical and sonographic risk factors have been reported. This study thus accurately determined the distance from the acromion to the greater tuberosity of the humerus using ultrasound. The primary objective of this study was to demonstrate that stroke type, supraspinatus thickness on the lesioned side, and BRS in patients with stroke were independent predictors of shoulder subluxation.

A positive correlation between shoulder subluxation and loss of motor function has been reported; BRS has been shown to be a significant predictor of GHS (9). A negative correlation between BRS and A-GT distance was observed (Spearman’s rho = −0.382). In contrast, a positive correlation was seen between BRS and supraspinatus muscle thickness on the lesioned side (Spearman’s rho = 0.184). The one-way ANOVA reported that the A-GT distance of BRS 2 was smaller than that of BRS 1 with a not statistically significant decreasing trend, possibly due to the mild increase of the shoulder muscle tone. Furthermore, the A-GT distance of BRS 3 and 4 was found to be significantly smaller than that of BRS 1, likely due to the increase in shoulder muscle tone during the period of common and separate movements. Finally, although no statistical significance was reported, the A-GT distance of BRS 5 was larger than that of BRS 4, possibly due to the decrease in muscle tone and the enlargement of joint space. In addition, in accordance with previous studies, BRS was proven to be a precipitating factor for hemiplegic shoulder subluxation in the multiple linear regression analysis (46). For every 1 grade increase in BRS, the AGT distance decreased by 0.102 cm, and the risk of subluxation decreased (*R*^2^ = 0.360, *p* < 0.01). One of the essential goals of stroke rehabilitation is upper limb motor control. Shoulder subluxation hinders motor recovery. We hypothesized that with an increase in BRS in post-stroke patients, the muscle tension of the rotator cuff muscle group gradually increases, pulling the humeral head into the glenoid, decreasing the A-GT distance, and reducing the risk of subluxation. More motor assessment tools should be used in the future, such as the B-stage (49), NIHSS item 5, Uppsala University Hospital Motor Assessment Scale (50), and the Motricity Index (51) to select the most suitable tool for anticipating shoulder subluxation.

The glenohumeral joint, which has the widest range of motion in the human body, is stabilized by muscles, ligaments, and other tissues known as dynamic stabilizers, including the rotator cuff muscles, long head of the biceps tendon, and other shoulder girdle muscles. Thus, severe arm paralysis due to stroke is accompanied by severe weakening of these dynamic stabilizers in the shoulder, which may decrease glenohumeral joint stability and alter the normal pattern of protective motion of the glenohumeral and scapulothoracic joints (13). According to an EMG study, the supraspinatus and posterior deltoid muscles resist shoulder subluxation (52). Our results demonstrated that the supraspinatus thickness was significantly reduced in the paralyzed compared sides with the non-paralyzed sides. The A-GT distance was significantly increased in the paralyzed sides compared with the non-paralyzed sides. In line with this result, using precise ultrasound evaluation, we found that supraspinatus muscle thickness on the lesioned side was a significant predictor of subluxation (*R*^2^ = 0.360, *p* < 0.01). A negative correlation was observed between the supraspinatus thickness on the lesioned side and the A-GT distance (Spearman’s rho = −0.474). The greater the thickness of the supraspinatus, the shorter the A-GT distance. The supraspinatus muscle is located in the supraspinatus fossa toward the lateral aspect of the acromioclavicular joint. It integrates into the glenohumeral joint capsule and terminates at the superior articular surface of the greater tuberosity of the humerus. The supraspinatus makes up the rotator cuff musculature and is involved in the dynamic stabilization of the glenohumeral joint. The onset of a stroke causes hemiplegic side muscle strength decreased, muscle tension became imbalanced, and muscle atrophy occurred (53). Especially supraspinatus muscles on paralyzed side. It does not contract well enough to resist the gravity of the shoulder joint and cause shoulder subluxation (14). Its application to the supraspinatus and posterior deltoid muscles in combination with conventional treatment was found to be more beneficial than conventional treatment alone in patients with hemiplegic shoulder subluxation (12, 54). In addition, further validation is needed regarding the relationship between the rotator cuff muscle groups, such as the posterior deltoid muscles, and shoulder subluxation.

It has been shown that 1/3 of patients with cerebral infarction experience shoulder subluxation, whereas the same is true for 1/2 of patients with cerebral hemorrhage (11). Similarly, we found that cerebral hemorrhage was more likely to cause shoulder subluxation than cerebral infarction in patients with 0.38 cm more A-GT distance (*R*^2^ = 0.360, *p* < 0.01). We hypothesize this to be a result of worse proprioception and slower recovery in patients with cerebral hemorrhage, which are important risk factors for shoulder subluxation. The results of the one-way ANOVA showed that patients with cerebral hemorrhage were more likely to have shoulder subluxation than those with cerebral ischemia, and the difference was statistically significant.

Although the results of the one-way ANOVA showed that females were more likely than males to experience shoulder subluxation, in the multiple linear regression results, sex did not act as an independent risk predictor. Although the thickness of the right supraspinatus muscle was greater than that on the left side (*p* < 0.01) and a tendency was seen for the A-GT distance on the right shoulder to be smaller than that on the left side, such difference was not statistically significant. Previous research indicates that humans are considered to be uniquely right-handed, with a species-wide ratio of right-handedness to left-handedness of 9:1 (55), which consistent supraspinatus thickness was greater on the right than on the left in our study. Thus, further studies are required in the future.

### Limitations

There are a few limitations that should be noted, including lack of dominant hand data, ambiguity in shoulder subluxation assessment and lack of differentiation of factors affecting the thickness of supraspinatus muscle. This study lacked information regarding the subject’s dominant hand as it was focused purely on the lesioned side and ignored the effect of handedness on the thickness of supraspinatus muscle. The lack of clear criteria for determining the presence or severity of shoulder subluxation by A-GT distance may be a major limitation in achieving the purpose of this study. There is a need for clear criteria to determine the presence and severity of shoulder subluxation on the future research. The factors that may affect the thickness of the supraspinatus muscle, such as decreased muscle tone, atrophy of the supraspinatus muscle brake muscle, and gravity pull of the upper limb during the soft paralysis period, were not differentiated in detail in this study, and further research is needed in the future.

## Conclusion

This study was the first to use an accurate and sensitive ultrasound technique to measure the A-GT distance to determine the risk factors associated with shoulder subluxation. We revealed that the main risk factors affecting shoulder subluxation included supraspinatus thickness on the lesioned side, stroke type, and BRS, as validated through multiple linear regression. In addition, supraspinatus thickness on the lesioned side measured using ultrasound may be a new index for predicting shoulder subluxation.

## Data Availability

The original contributions presented in the study are included in the article/supplementary material, further inquiries can be directed to the corresponding author.
